# Blast-Associated Shock Waves Result in Increased Brain Vascular Leakage and Elevated ROS Levels in a Rat Model of Traumatic Brain Injury

**DOI:** 10.1371/journal.pone.0127971

**Published:** 2015-05-29

**Authors:** Shushi Kabu, Hayder Jaffer, Marianne Petro, Dave Dudzinski, Desiree Stewart, Amy Courtney, Michael Courtney, Vinod Labhasetwar

**Affiliations:** 1 Lerner Research Institute, Department of Biomedical Engineering, Cleveland Clinic, Cleveland, Ohio, United States of America; 2 Lerner Research Institute, Medical Device Solutions, Cleveland Clinic, Cleveland, Ohio, United States of America; 3 BTG Research, Colorado Springs, Colorado, United States of America; Hungarian Academy of Sciences, HUNGARY

## Abstract

Blast-associated shock wave-induced traumatic brain injury (bTBI) remains a persistent risk for armed forces worldwide, yet its detailed pathophysiology remains to be fully investigated. In this study, we have designed and characterized a laboratory-scale shock tube to develop a rodent model of bTBI. Our blast tube, driven by a mixture of oxygen and acetylene, effectively generates blast overpressures of 20–130 psi, with pressure-time profiles similar to those of free-field blast waves. We tested our shock tube for brain injury response to various blast wave conditions in rats. The results show that blast waves cause diffuse vascular brain damage, as determined using a sensitive optical imaging method based on the fluorescence signal of Evans Blue dye extravasation developed in our laboratory. Vascular leakage increased with increasing blast overpressures and mapping of the brain slices for optical signal intensity indicated nonhomogeneous damage to the cerebral vasculature. We confirmed vascular leakage due to disruption in the blood-brain barrier (BBB) integrity following blast exposure. Reactive oxygen species (ROS) levels in the brain also increased with increasing blast pressures and with time post-blast wave exposure. Immunohistochemical analysis of the brain sections analyzed at different time points post blast exposure demonstrated astrocytosis and cell apoptosis, confirming sustained neuronal injury response. The main advantages of our shock-tube design are minimal jet effect and no requirement for specialized equipment or facilities, and effectively generate blast-associated shock waves that are relevant to battle-field conditions. Overall data suggest that increased oxidative stress and BBB disruption could be the crucial factors in the propagation and spread of neuronal degeneration following blast injury. Further studies are required to determine the interplay between increased ROS activity and BBB disruption to develop effective therapeutic strategies that can prevent the resulting cascade of neurodegeneration.

## Introduction

The frequent use of improvised explosive devices makes blast injury a constant threat for military troops deployed in areas of conflict. Blast-associated shock wave-induced traumatic brain injury (bTBI) is known to manifest itself as mild to severe physiological and psychological deficits, and often leads to post-traumatic stress disorder [1]. It is now recognized that a significant number of combat injuries diagnosed in returning veterans are linked to bTBI [2]. From initial diagnosis, to progression over time and eventual outcome, the nature of bTBI is varied and heterogeneous, presenting itself in dissimilar physical characteristics and resultant neuronal consequences [3]. The current system used for categorizing blast injuries is based on the nature of the initial mechanical insult, thus classifying the resulting injury into any of the four types of bTBI: primary (caused by the direct effect of a blast wave), secondary (caused due to the physical contact of the victim with blast debris and fragments), tertiary (occurring when the victim is physically displaced by the blast wave and winds), or quaternary (resulting from chemical and thermal damage caused by the blast) [3]. Although the exact transduction pathway that causes bTBI remains unknown, probable mechanisms include **a)** direct passage of the blast wave via the skull, causing mechanical disruptions, acceleration and/or rotation of the head and **b)** indirect passage of the blast wave though the vascular system, resulting in propagation of localized high pressures (“overpressures”) and/or sudden disruptions to the blood flow [4,5].

Pathophysiological mechanisms associated with blast injuries include primary damage to the brain involving both direct initial insult from the blast wave and subsequent activation of secondary pathogenic cascades that collectively influence the temporal progression of the primary insult [4,6,7]. The kinetic energy transfer from the blast wave to the brain is believed to be responsible for initiating diffuse axonal injury and secondary injury cascades [8,9], leading to cognitive deficits that develop and become apparent with time [10,11]. Neuronal, axonal and glial injury, have also been reported to manifest diffusely over time [12,13]. Commonly used animal models of traumatic brain injury include controlled cortical impact, weight drop, vacuum deformation and fluid percussion, and less common models use pendulums, darts or bolts to induce damage [14,15]. Although direct impacts and penetrating injuries are disposed to cause more focal and pronounced damage, TBI from exposure to the blast waves is known for its distinctive diffuse injury pattern, either localized or widespread in scope [16]. bTBI often manifests itself as an invisible injury, as current radiological/imaging techniques are unable to diagnostically discern damage in an affected individual.

A broad classification of the currently available investigational models of bTBI induced by blast tubes comprises compressed gas-driven shock tubes (that employ air, nitrogen or helium) or detonation-driven shock tubes (that use chemical explosives) to produce blast waves [17–20]. The objectives of this study were to **a)** design a laboratory-scale shock tube that mimics blast-associated shock waves and **b)** validate and analyze the effects of the resulting blast-induced injuries on the rat brain. Our data show that this shock-tube design is capable of replicating blast waves, as seen in battlefield conditions, by providing a repeatable control of the intensity, complexity, and duration of the shock-wave profiles. Our data show increased brain vascular leakage and reactive oxygen species (ROS) formation with increasing blast pressure, resulting in sustained neuronal injury as evident from astrogliosis and cell apoptosis.

## Materials and Methods

### Shock-Tube Design

**Fig 1:**
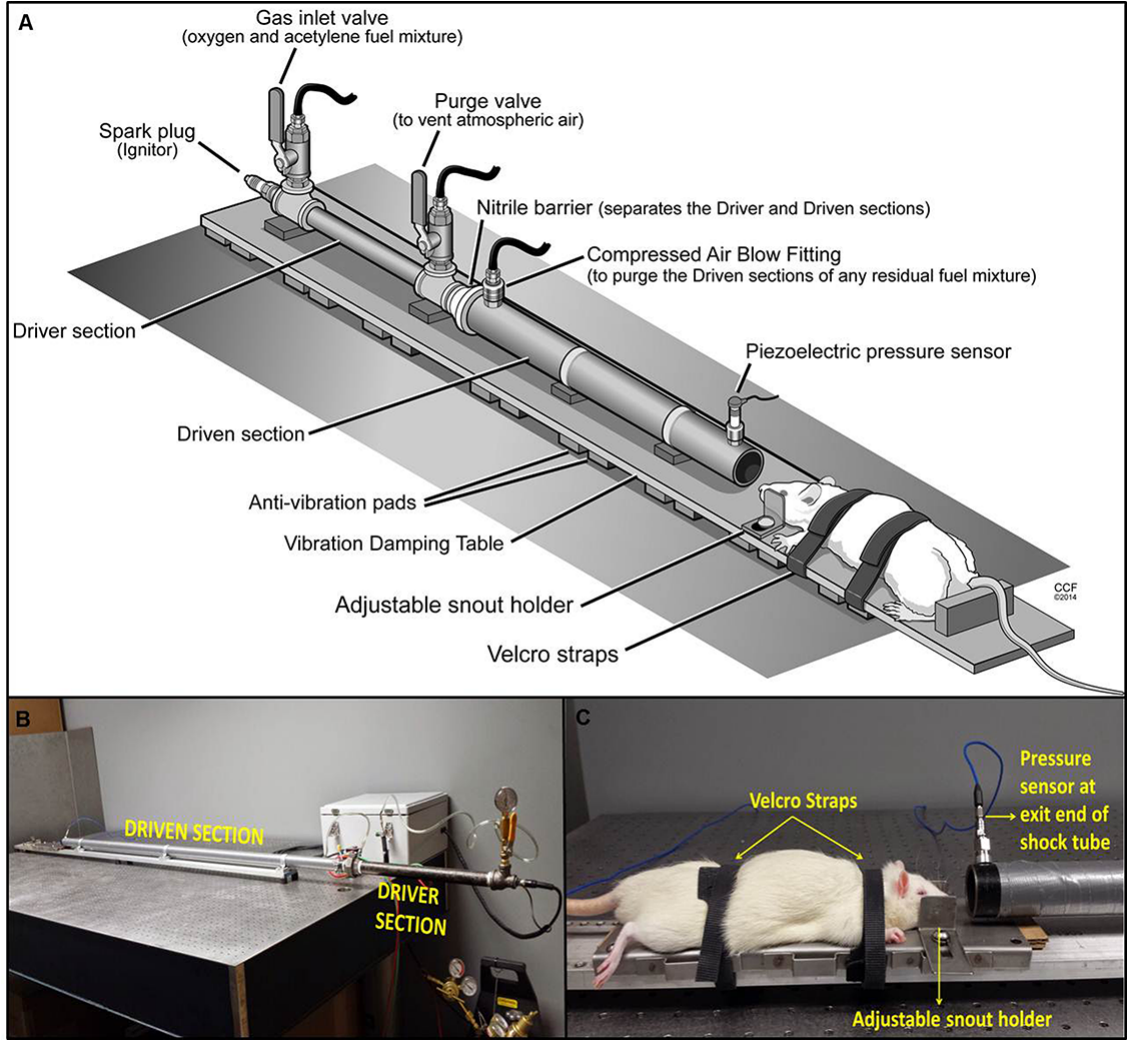
Shock-tube design and experimental setup for bTBI. **A)** Design and construction of oxy-acetylene shock tube. The shock tube comprises of the driver and driven sections, separated by a nitrile barrier, where the driver section is filled with the fuel mixture for 50 s though the gas inlet valve, while the purge valve allows the ambient atmospheric air to escape. Once filled, the driven section is blown out with compressed air via the compressed air blow fitting to eliminate any residual fuel mixture that may have leaked into the driven section while filling. On being triggered by the software, the spark plug ignites the fuel mixture to form the blast wave. **B)** Actual experimental setup of the shock tube. **C)** Animal and sensor placement. The anesthetized rat is placed on the rat placement holder at a distance of 3 cm from the exit end of the blast tube and strapped with two Velcro straps across the head and back, respectively, to prevent bulk motion. The adjustable snout holder also helps to keep the animal stable during the blast exposure.

The shock tube used in this study was modeled based on Drs. Courtney’s oxy-acetylene based shock tube design as previously published [21]. This design provides realistic blast wave profiles, has minimal jet effect and requires no specialized equipment or facilities. However, so far it has been used solely for material testing, but never for animal studies. To develop an animal model of bTBI, several modifications were made to the previous shock tube design, for easy adaptability in a laboratory setting. The shock-tube design comprises the driver and driven sections; the driver section is filled with the fuel mixture and the driven section holds air at atmospheric pressure (Fig 1A and 1B). A stoichiometric mix of oxygen and acetylene serves as the oxy-fuel mixture for the production of a shock wave. The driver and driven sections are fabricated from commercially available standard steel pipes (Schedule 40, 0.3 inch thickness, ordered from McMaster-Carr, Aurora, OH) and wrapped with an industrial grade duct tape (3M Corp., St. Paul, MN) to minimize vibrations following a blast. A thin nitrile finger cot (3 mils thickness, ordered from McMaster-Carr) serves as a barrier to separate the driver and driven sections. An automated spark plug (Champion RZ7C nickel spark plug, Sun City, CA), driven by an automatic coil running on a 9V battery, is attached to the ignition-end face of the driver to ignite the fuel mixture when triggered externally. A custom-designed LabVIEW 2011 program (National Instruments, Austin, TX) controls the automated and precise release of oxygen and acetylene gas, each via a solenoid valve, and also triggers the spark plug to ignite the fuel mixture. A piezoelectric high-speed pressure sensor (PCB 102B15, Piezotronics, Depew, NY) for dynamic pressure measurements is mounted at the end of the driven section to measure the pressure profile of the generated blast wave near the shock-tube opening. Blast-wave characteristics were measured for a combination of driver configurations with a single driven section (Table 1), to attain varying blast pressure intensities and to classify the obtained pressure ranges into mild, moderate, and severe exposures.

**Table 1:** Effect of driver dimensions (length and diameter) on pressure range and mean pressures.

Driver section length (in)	Driver section diameter (in)	Driven section diameter (in)	Measured pressure range (psi)	Mean pressure ± s.e.m. (psi)
1	1	1	45–47	45.7 ±0.3
6	1	1	65–69	67.4 ±0.5
10	1	1	85–90	87.9 ± 0.6
12	1	1	97–103	99.9 ± 0.8
18	1	1	119–126	121.0 ± 0.7
10*	0.5	1	17–45	34.5 ± 9.54

Data as shown as mean± s.e.m., *n* = 10. Driven section length: 72.5 in.

Mild (>20–50 psi), Moderate (>50–90 psi) and Severe (>90–130 psi) bTBI classification.*This length was from the first-generation design of the shock tube, in which the driver was filled for variable time intervals (range, 500 ms-3 s) to produce an incremental increase in pressures (range, 17–44 psi). Abbreviations: in-inch; psi-pounds per square inch.

### Operation

The shock-tube apparatus was placed on a vibration-isolation table (Technical Manufacturing Co., Peabody, MA). The driver section was filled for 50 s (same for all driver configurations) with a mixture of oxygen and acetylene though the gas inlet valve while the purge valve, which functions in an ON-OFF manner, was left open during filling to allow air in the driver section to escape. This avoided the mixing of oxy-fuel mixture with air, which we noticed can cause variations in blast intensity and overpressures. Gas inlet and purge valves were closed once the driver section was filled with the gas mixture. As a precaution, the inside of the driven section was blown out with compressed air through the compressed air blow fitting prior to ignition, to purge any fuel mixture that may have leaked out into the driven section during filling. This step prevents a deflagration-to-detonation type explosion from occurring. Once triggered, the spark plug ignited the fuel mixture and caused the driver gases to expand rapidly and rupture the nitrile membrane, forming the blast overpressure wave as it travels along the driven section.

### Waveform acquisition and analysis

The blast overpressure wave was recorded at a sample rate of 0.5 MHz via the piezoelectric sensor (PCB 102B15) at the end of the driven section. Analog output from the transducer was converted to a digital signal using a National Instruments USB-5132 converter. The pressure transducer was also synced to the LabVIEW program, which acquired the digitized data and displayed the final readout as a pressure-time profile of the shock wave. The effects of various driver dimensions on the pressure-time profiles of the blast overpressure wave were fully validated and characterized.

### Animal Study

The Institutional Animal Care and Use Committee (IACUC) of Cleveland Clinic approved the study. All procedures followed the guidelines established by the IACUC of Cleveland Clinic. Adult 3- to 4-month-old male Sprague-Dawley rats (Harlan Laboratories, Indianapolis, IN) weighing 300–400 g were used. The animals were housed with free access to food and water in a 12-h dark/12-h light cycle. Animals were anesthetized using 2.5–3.5% isoflurane. A lubricating eye ointment with an aluminum foil shield was placed over the eyelids to reduce the likelihood of gross optical injuries. The rat was placed on the rat placement holder at a distance of 3 cm from the exit end of the blast tube, with the head facing the blast tube, and strapped with two Velcro straps across the head and back respectively to prevent bulk motion (Fig 1A and 1C). The adjustable snout holder also helps in keeping the rat in position during the blast exposure. Animal placement from the exit end of the shock tube was selected subjectively, keeping in consideration that the animal does not block the flow of the burned gases/air after the blast, while also preventing the blast wave from decaying its Friedlander profile. Since animals were exposed head-on to the blast exposure (Fig 1), there was no need for a shield to protect thorax and abdomen. In few other shock tube designs, animals are placed inside the tube where such shielding is necessary [20]. Following blast exposure, animals were allowed to recover from anesthesia and were monitored for time taken to regain consciousness. Animals were also assessed for any signs of pain, distress, and difficulty in breathing post-blast exposure.

### Evaluation of BBB leakage following exposure to different blast intensities

We used a recently developed sensitive optical imaging method from our laboratory to evaluate BBB leakage by measuring the fluorescence of Evans Blue (EB) dye in the brain. This imaging method, with 1000-fold greater sensitivity than the conventional ultraviolet (UV) spectrophotometric method, allows for quantification of vascular leakage in various areas of the brain. In our previous studies, we validated our optical imaging method by comparing it to conventional UV spectrophotometry method [22]. There was a direct co-relation between the amounts of EB dye determined by both the methods. Further, the tissue background signal is insignificant and the optical signal intensity from the brain slices correlates to the amount of EB dye [22]. Thus one can quantitatively determine the amount of Evans blue dye leaching out in the brain under different blast conditions by measuring the optical signal intensity from the brain slices. Using this method, we analyzed vascular leakage 24 h post-blast in animals exposed to various blast intensities, falling within the mild, moderate and severe exposure ranges, and compared it with naïve controls that did not receive a blast exposure. For this step, an EB dye solution (4% w/v in saline) was injected (2.5 ml/kg) intravenously though the tail vein 24 h post-blast exposure. Animals were euthanized 2 h after the EB dye injection and transcardially perfused with heparinized saline (10 U/ml) to remove blood and EB dye from the circulation. Harvested brains were imaged using the Maestro Imaging system (Perkin Elmer, Waltham, MA), as described in our previous study [22]. The blue filter was set between wavelengths 500 nm and 720 nm with 787 ms exposure time to capture any autofluorescence and provide an outline of the sample; the near-infrared filter was set between 740 nm and 950 nm with 1183.04 ms exposure time to visualize the EB dye. Whole brains were sectioned coronally into seven 2-mm-thick slices, rostral to caudal, using a brain matrix. The total signal intensity from all brain slices together was measured using Maestro and the result was normalized per pixel. These values were then used to calculate the amount of EB dye from the standard plot prepared in brain tissue homogenate with different amount of EB and signal intensity as per the protocol described in our previous study [22]. For EB dye mapping purposes, we compared different anatomical areas of the severely injured brain (120–125 psi) and measured signal intensity in selected regions of interest in comparison to naïve rats that were not exposed to a blast.

The BBB disruption was further confirmed immunohistochemically by analyzing the extravasated immunoglobulin G (IgG) in the brain sections [23–25]. For this, harvested brains were kept in 10% normal buffered formalin at room temperature (RT) for 24 h, and later moved to PBS and kept at 4°C until standard processing and embedding in paraffin. Coronal and axial sections (5 μm) were cut from the blast-injured and naïve control brains. Sections were deparaffinized and hydrated, and antigen retrieval was performed with two repetitions of Trilogy pretreatment solution (Cat. No. CMX 833 1CS; Cell Marque, Rocklin, CA) for 30 min each in a conventional food steamer. Slides were cooled down to RT, rinsed in tap water and put in 0.3% H_2_O_2_ in 80% methanol for 20 min to block endogenous horseradish peroxidase (HRP) activity. Sections were incubated in blocking solution for 1 h at room temperature to minimize non-specific binding. Tissue sections were then stained with fluorescein (FITC)-conjugated AffiniPure Donkey Anti-Rat IgG secondary antibody (1:200, Jackson Immunoresearch, West grove, PA) and later mounted using a glass coverslip and Vectorshield mounting medium containing DAPI to visualize nuclei (Vector Labs, Bulingame, CA). Slides were scanned in a Leica SCN400 scanner (Leica Biosystems, Buffalo Grove, IL) at 20X magnification.

### Edema formation following severe blast exposure

To determine the amount of cerebral edema, at 3 h post-exposure (severe blast range, 120–125 psi), brains were harvested without transcardial perfusion and weighed (wet weight) immediately. Whole brains were then dried in an oven at 70°C for 48 h and weighed again (dry weight). This is the most commonly used method to determine brain edema, although there are few more sensitive but complex methods to analyze brain water content [26]. The percentage of water in the brain was calculated ([wet weight-dry weight)/wet weight] x100) to compare the water content with respect to naïve controls where animals were not exposed to blast waves.

### ROS formation following blast exposure

The harvested brains were sectioned coronally into seven 2-mm-thick slices, rostral to caudal, using a brain matrix and incubated in a solution (5μmol/L) of CellROX deep red reagent (Life Technologies, Grand Island, NY) in an incubator maintained at 37°C for 60 min. This assay works on the conversion of colorless dye to deep red fluorescence in the presence of ROS. The brain sections were washed with phosphate buffered saline (1X) thee times and immediately imaged using the Maestro Optical Imaging System to capture the fluorescence signal. Maestro’s blue filter was set between 500 nm and 720 nm wavelengths for image acquisition, with 800 ms exposure time. The yellow filter was set between wavelengths of 630 nm and 800 nm, with 800 ms exposure time. The blue filter captures any autofluorescence and provides an outline of the sample; the yellow filter visualizes the fluorescence coming from the CellROX reagent. The total signal intensity from all brain slices together was measured, and the results were normalized per pixel to calculate and compare the relative ROS activity between rats exposed to the blast waves (mild or severe in impact) at various time points (24, 48 and 72 h) and naïve control groups. We found this assay to be more sensitive than other ELISA methods conventionally used to determine by-products of oxidative stress, and able to capture signal from all brain slices, thus representing the global change in ROS activity [27,28].

### Immunohistochemical assessments of brain damage following blast exposure

We conducted immunohistochemical (IHC) studies to evaluate apoptosis and astrocytosis after mild blast exposures (20–25 psi), and to validate the BBB leakage ensuing post-blast using an alternative method. Harvested brains were kept in 10% normal buffered formalin at room temperature (RT) for 24 h, and later moved to PBS and kept at 4°C until standard processing and embedding in paraffin. Coronal and axial sections (5 μm) were cut from the blast-injured and naïve control brains. Sections were deparaffinized and hydrated, and antigen retrieval was performed with two repetitions of Trilogy pretreatment solution (Cat. No. CMX 833 1CS; Cell Marque, Rocklin, CA) for 30 min each in a conventional food steamer. Slides were cooled down to RT, rinsed in tap water and put in 0.3% H_2_O_2_ in 80% methanol for 20 min to block endogenous horseradish peroxidase (HRP) activity. After rinsing with water, slides were incubated with primary antibody (diluted in Antibody Diluent with Background Reducing Components (S3022; Dako) for 60 min at RT in a humid chamber and then rinsed three times for 5 min each in PBS. 10 min incubation with HRP-labeled Rb SuperPicTure polymer (SuperPicTure Polymer Detection Kit, Cat. No. 87–9263; Invitrogen, Life Technologies, Grand Island, NY) was used for rabbit primary antibodies. Slides were rinsed twice for 5 min in PBS and the targeted markers were then visualized with the HRP substrate diaminobenzidine (DAB) prepared as instructed in Invitrogen’s kit (Cat. No. 87–9999). Slides were counterstained with either hematoxylin or eosin to visualize non targeted cell structures. Markers investigated were caspase-3 for apoptosis and GFAP (glial fibrillary acidic protein) for astrocytes. Slides were scanned in a Leica SCN400 scanner (Leica Biosystems, Buffalo Grove, IL) at 20X magnification. Primary antibodies used to target these markers were: Rb polyclonal anti-GFAP (Dako Code No Z 0334, dil.1:500); Rb polyclonal anti-Active Caspase3 (R&D Systems, Cat. No. AF835, dil.1:200).

### Statistical Analysis

All numerical data are expressed as mean ± standard error of mean (s.e.m.). Statistical significance was determined using one-way ANOVA with Newman-Keuls multiple comparison test (significance level- 0.05) to compare EB leakage, ROS activity levels between groups and at various time points. Statistical significance was calculated using one-tailed paired t-test (confidence intervals- 95%) to compare edema between naïve and blasted groups. A *p* value of ≤ 0.05 was considered statistically significant for all data.

## Results

### Optimization of shock tube design and resulting blast intensities

**Fig 2:**
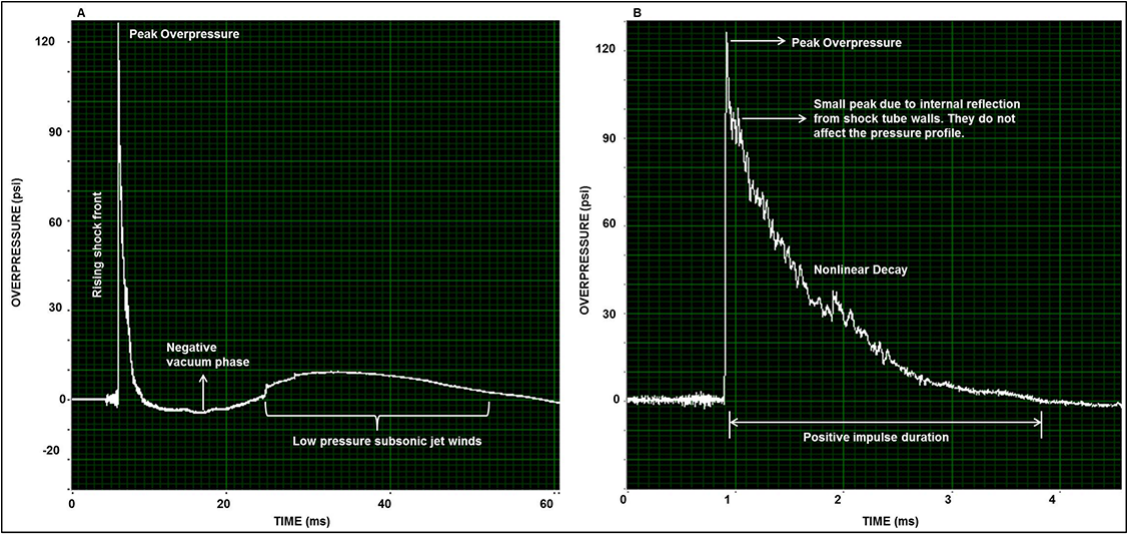
Typical pressure-time profiles of the shock wave form. **A)** Overview of the entire shock-wave profile. Pressure-time profiles of blast waves generated by different driver section configurations were typical to the Friedlander waveform of free-field blast waves. **B)** Detailed description of the peak overpressure, period of nonlinear decay, and positive pulse duration. A typical shock waveform lasted for 2–4 ms, depending on the driver length, and was composed of a steep shock front of positive overpressure, followed by a period of exponential decay with a negative vacuum phase.

The pressure-time profiles of the shock waves generated by different driver section configurations were typical to the Friedlander waveform of free-field blast waves (Fig 2A). A typical shock waveform lasted for 2–4 ms, depending upon the driver length. For example, an 18 inch driver resulted in 3.8 ms of positive overpressure duration whereas shorter driver (6 inch) resulted in 2 ms. The waveform was composed of a steep shock front of positive overpressure, followed by an exponential decay with a negative vacuum phase (Fig 2B). The Friedlander nature of the shockwave, short positive pulse duration of 2–4 ms, and extensive array of peak pressures of 20–130 psi conform to clinically relevant shock waves produced due to explosion of improvised explosive devices (IEDs) commonly used in the battle field, which produce shock waves in the range of 7.25 psi to 145.03 psi with exposure duration of 2–10 ms [29].

**Fig 3:**
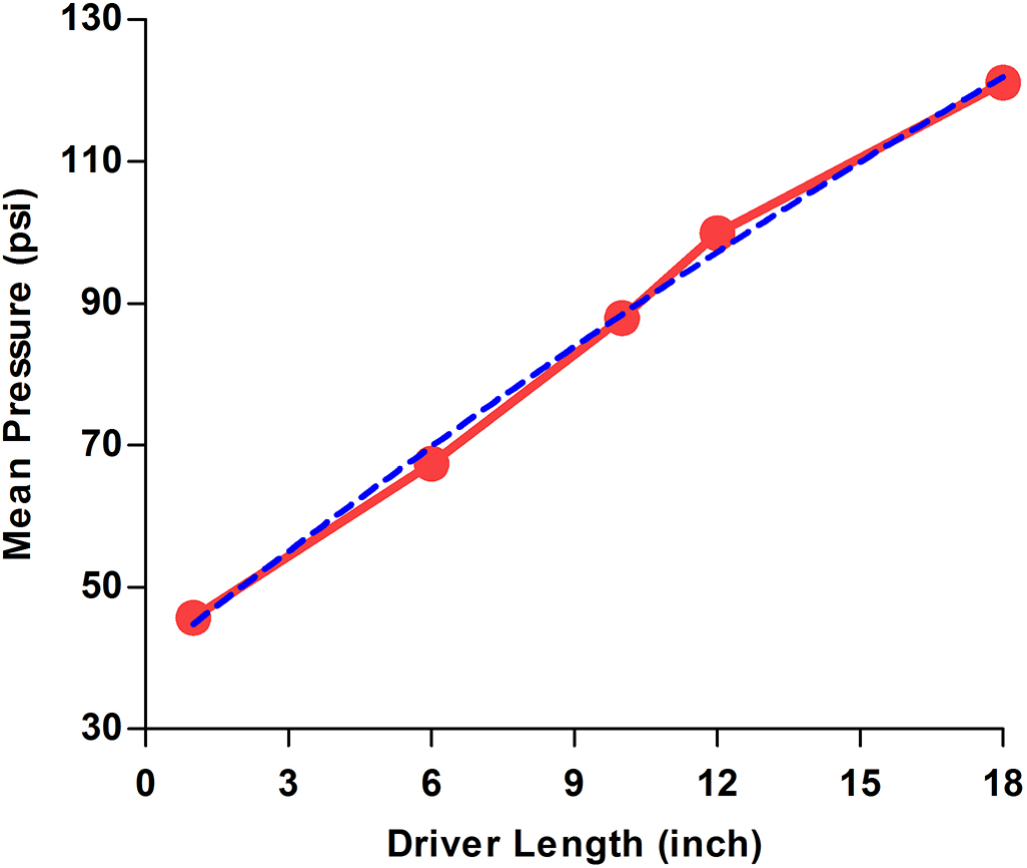
Relationship between mean pressures produced and driver length. Mean peak overpressures (in red) produced by drivers of various sizes, with s.e.m less than 1% (s.e.m. bars are within the symbols). Increasing the driver length led to an increase in peak overpressures (range: 40–130 psi). Regression curves (in blue) were obtained on the acquired data using second order polynomial regression and R^2^ = 0.99. The generated pressure waves and mean peak pressures for each driver size were consistent, and the resulting intensities were labeled as mild (>20–50 psi), moderate (>50–90 psi) and severe (>90–130 psi) blast exposures. Data are shown as mean ± s.e.m., *n* = 10 for each driver configuration.

Characterization of various driver configurations showed that increasing the driver length leads to an increase in peak overpressures (range: 40–130 psi), showing an almost linear correlation (R^2^ = 0.99) between driver length and mean peak pressures. The generated pressure waves and mean peak pressures for each driver size were consistent and reproducible (Table 1; Fig 3). Thus the resulting blast intensity ranges were classified as mild (>20–50 psi), moderate (>50–90 psi) and severe (>90–130 psi) blast exposures. Polynomial regression analysis performed on the obtained data was in congruence with our findings (Fig 3). Interpolation of the regression curve helps us to predict and estimate the mean pressures, which can be expected from drivers that fall within the range of driver lengths we have currently tested.

In the first-generation shock tube, the driver (17.5 in length, 0.5 in diameter) was filled for a preset time interval (range, 500 ms—3 s), and no venting or purging was conducted. However, inconsistencies of ±10 psi among similarly timed fill shots were observed in the obtained pressures (Table 1). These irregularities in peak pressures prompted us to modify the design by introducing a purge valve and compression blow fitting in the second-generation shock tube; this modification has prevented any accidental fuel mixture leakage and produced reproducible and consistent blast overpressures.

As previously noted by Courtney et al [21], thermal damage from the shock tube is not likely, even though the combustion temperature of oxy-acetylene is high. Prior to ignition, the fuel mixture rests at an ambient pressure within the driver section. The molar volume of the combustion products is smaller than the molar volume of the reactants. On ignition, burn products do not leave the tube and the test subject is only exposed to the air blast from the air that was in the driven section of the shock tube which is at room temperature prior to the shock wave passing through it. There is no large expansion of the fuel mix or a flame emerging from the driven section. The gaseous combustion products start to lose heat as they travel down the driven section of the shock tube, and the contact time between the combusting gases and the test object is very short causing nominal heat transfer. Dr. Courtney’s conducted tests where paper, plastic, leather and bone were placed 1–3 cm from the shock tube opening, and no evidence of thermal damage (such as melting, singeing or discoloration) was observed even after several exposures [21].

### Brain Injury

#### Physiological/Biological Response

Immediately after the blast exposure, the overall breathing pattern and rhythm changed in all injury groups. Rats seemed incapacitated and the respiration rate was slow and deep for 1–2 minutes post-blast followed by a smooth, slow recovery within 3–5 minutes. Once awake, rats seemed limp, tired and sluggish with poor appetites. Seizure like twitching was also observed in the moderate and severe bTBI study groups for almost up to 3–6 hrs.

#### Increased vascular leakage with increasing pressure intensities

**Fig 4:**
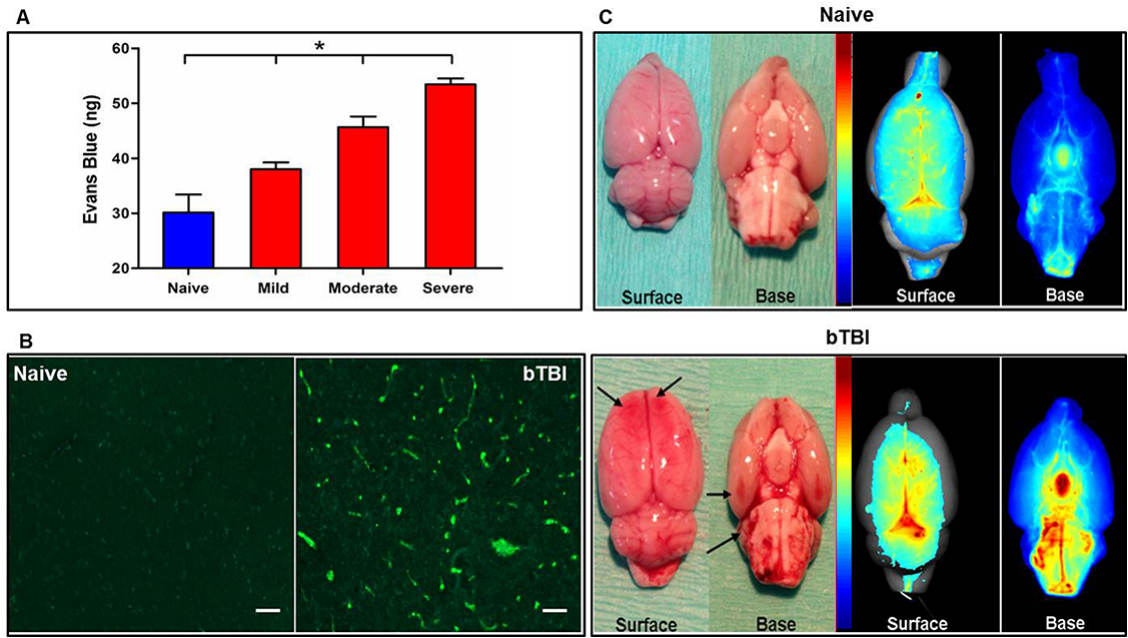
Effect of varying blast intensities on vascular leakage and BBB damage. Animals were exposed to mild (>20–50 psi), moderate (>50–90 psi) and severe (>90–130 psi) blast pressures. The damaged brains were harvested and imaged at 24 h post injury. **A)** A commensurate increase in the amount of EB dye leakage in brain tissue (in nanograms) and intensity of the shock waves used, i.e., mild (>20–50 psi), moderate (>50–90 psi) and severe (>90–130 psi). Data are shown as mean ± s.e.m., *n* = 4; *p ≤ 0.05. Statistical significance was calculated one-way ANOVA with Neuman-Keuls post-test. **B)** Representative images of immunofluorescently detected extravasated IgG in bTBI vs. naïve brains. Photo micrograph displays the increased IgG immunoreactivity in a region from the striatum of mild-bTBI brain as evidenced by enhanced fluorescent signal intensity. Absent leakage indicates integrity of the BBB as is evident in the naïve brain section which displays almost no fluorescent signal. Scale bar: 50 μm. **C)** Gross brain pathology following bTBI showed larger blood vessel diameters and hematomas (black arrows), more prominently in the frontal and occipital lobes (left side). Optical images of EB dye leakage shows prominent leakage in the brainstem area in bTBI animals.

Quantification of signal intensity using optical imaging method shows increased EB dye leakage with increased peak blast pressures. Thus we classified the resulting injuries into mild (>20–50 psi), moderate (>50–90 psi) and severe (>90–130 psi) bTBI (Fig 4A). Our categorization of mild, moderate and severe bTBI was based on the range of pressures used and the subsequent vascular leakage produced; however, further analysis of the injury response at different pressure intensities is needed to affirm this classification.

In the control group, animals did not undergo any surgery or blast exposure, except a tail vein injection for the EB dye. The controls did not show any significant signal from the EB dye except in brain regions lacking the BBB, such as choroid plexus and circumventricular organs [30]. Similar results have been obtained from our previously published work on optical imaging of BBB [22]. An increase in the total amount of EB dye leakage from all seven slices combined correlated positively with increasing pressure intensities of the blast waves to which the animals had been exposed (Fig 4A). Increased IgG extravasation was also observed, specifically in the hippocampus, thalamus and striatum near the ventricles, indicating increased BBB permeability following blast exposures (Fig 4B). Gross brain observation following severe blast exposure revealed the presence of enlarged blood-vessel diameters and hematomas, prominently in the frontal lobe, occipital lobe, and brainstem areas (Fig 4C). We saw no evidence of an injury or marked changes in the brain tissue at pressures below 20 psi.

#### Mapping of vascular leakage and edema formation

**Fig 5:**
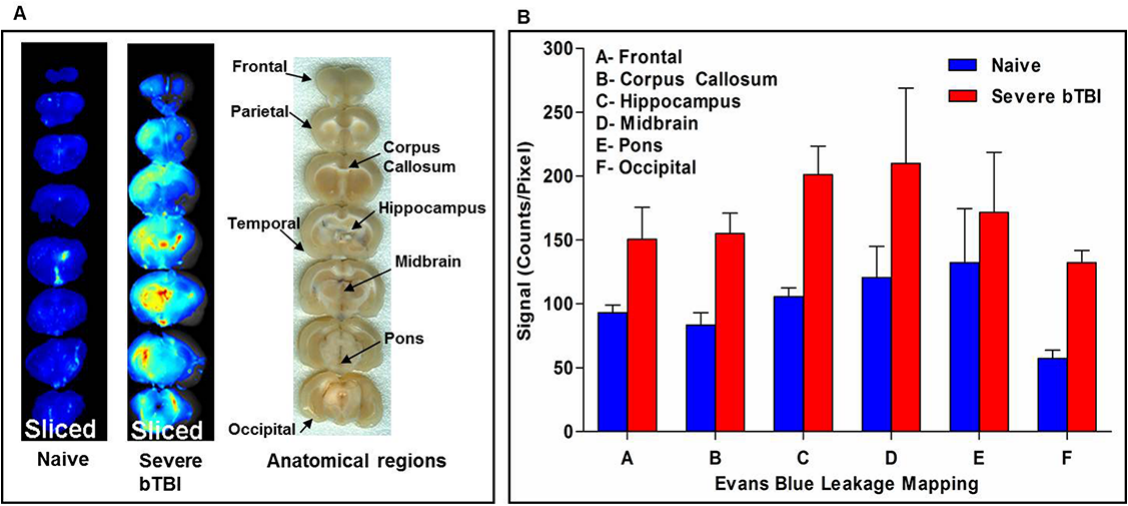
Mapping of vascular leakage in severe bTBI and naïve controls. For quantitative mapping of EB leakage, the entire brain was sliced into seven 2 mm thick sections. The brain sections were imaged at 24 h post-injury. **A)** Brain slices (left columns) represent optical images of brain tissue sections from severely injured group depicting an increase in EB dye leakage in the periventricular area (corpus callosum and hippocampus) and the occipital lobe. In control animals (without bTBI), EB dye leakage is seen in the choroid plexus and circumventricular organs, where there is no BBB. An actual photograph (right column) of the sliced brain sections (taken using HP Scanjet G4010) showing the different anatomical areas of interest. **B)** Mapping of EB dye leakage comparing naïve control and severe bTBI (120–125 psi) brains from six different anatomical areas: frontal lobe, corpus callosum, hippocampus, mid brain, and pons.

**Fig 6:**
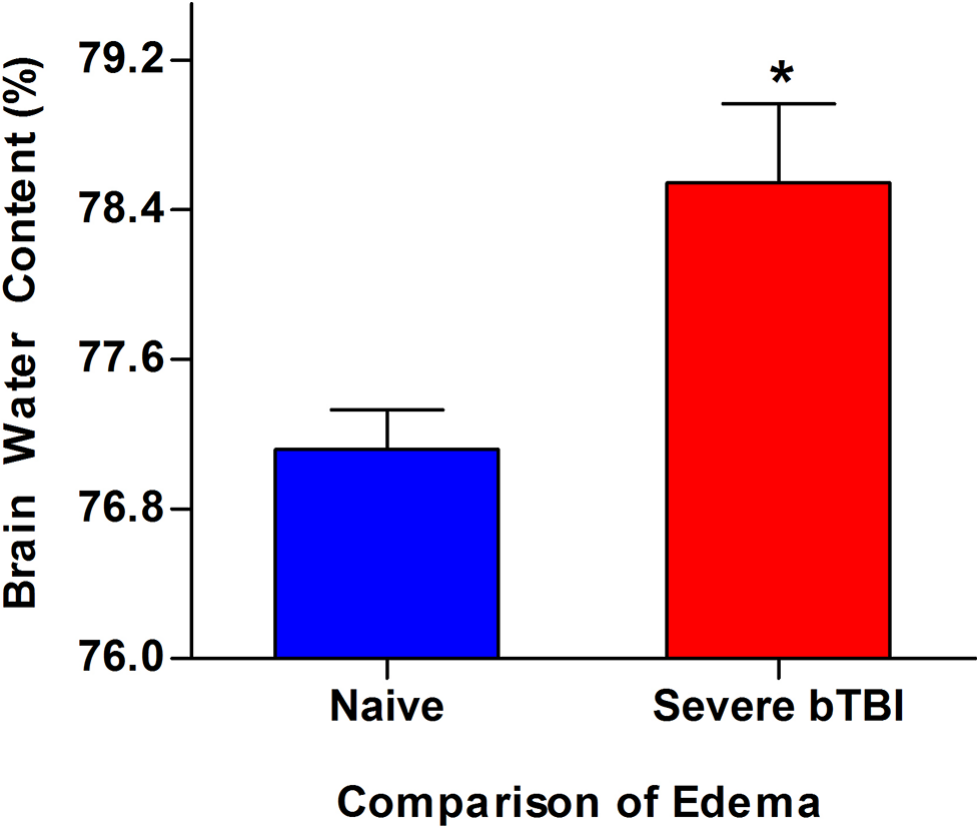
Edema formation in severe bTBI and naïve controls. Animals were exposed to severe (120–125 psi) blast pressures and brains were harvested 3 h post-exposure. After excision, the damaged brains were dried for 48 h at 70°C and later weighed. Data are shown as mean ± s.e.m., *n* = 3,*p = 0.048. Statistical significance was calculated using one-tailed paired t-test with 95% confidence intervals.

Gross observations showed that certain areas of the brain exhibited greater vascular leakage than others. Further brain examination by Maestro for optical signal intensity due to EB dye extravasation showed an increased and diffuse vascular leakage (red = high fluorescent signal, blue = low fluorescent signal) compared with brains of naïve animals (Fig 5A). With our optical imaging method, it is feasible to quantitatively map the extent of vascular leakage in different brain areas. The optical signal intensities for different regions of the brain were measured from the brain slices shown in Fig 5A to quantitatively determine vascular leakage in different areas of the brain (Fig 5B). Fig 5A (right column) are the regular scanned images (taken using HP scanner) of brain slices to indicate the respective areas of the brain that were quantified for mapping using optical imaging. The percent increase in vascular leakage was calculated by measuring the difference in total signal intensity averages in severe blast-exposure group versus naïve controls. The anatomical areas of the brain that showed leakage, in an increasing order, were as follows (percent increase): occipital lobe (131%), hippocampus (91%), corpus callosum (86%), midbrain (74%), frontal lobe (62%) and pons (30%). Based on varied signal intensities, it was evident that BBB damage is not anatomically uniform and that certain brain areas are more susceptible to vascular leakage than others (Fig 5A and 5B). Rats exposed to severe bTBI also demonstrated about a ~2% increase in edema as compared to naïve brains in 24 h post-injury (Fig 6), further supporting the above vascular leakage data.

#### Increased ROS activity with increasing blast pressures and at different times

**Fig 7:**
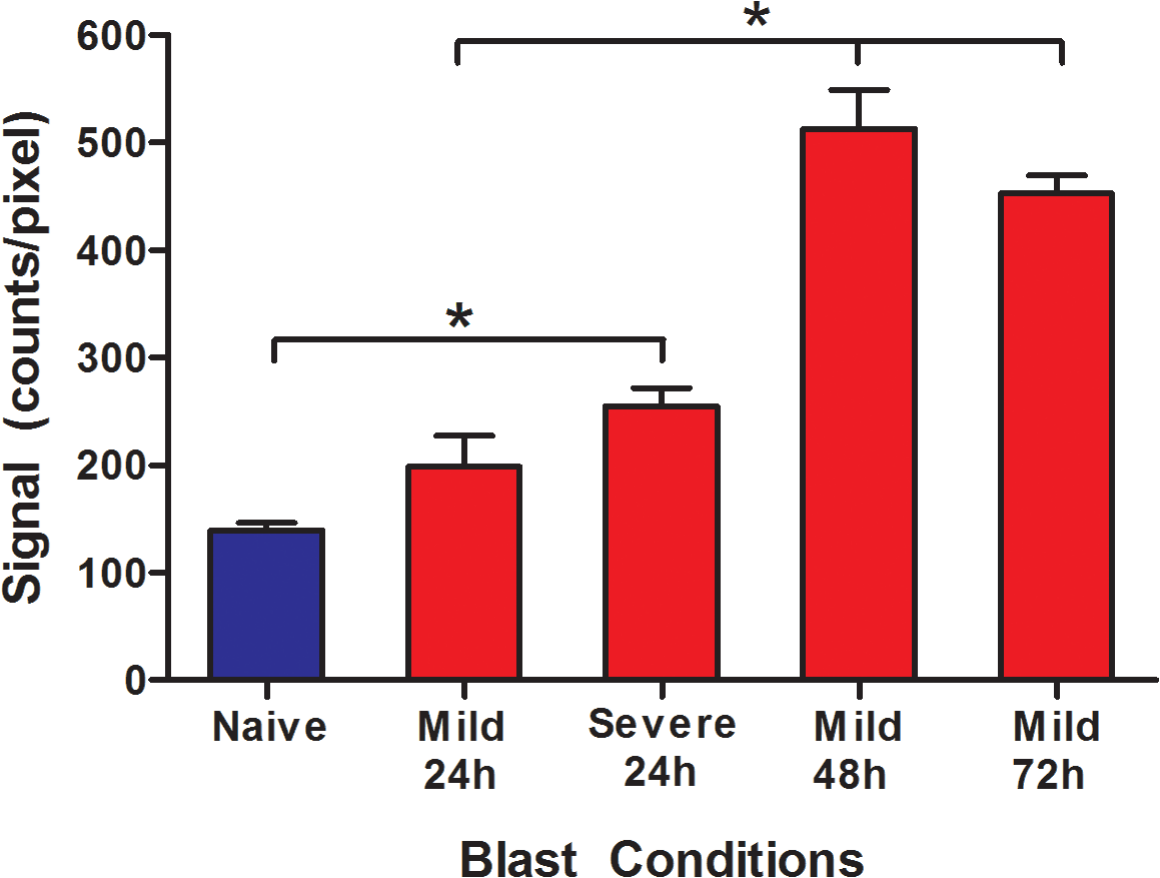
Changes in ROS activity with blast conditions and with time following blast exposure: Animals exposed to different blast pressures and with time following blast exposure at mild condition were analyzed for ROS levels. The conversion of colorless CellROX™ dye to deep red fluorescence in the presence of ROS was captured using the Maestro Optical Imaging System. The total signal intensity from all brain slices together was measured, and the result was normalized per pixel to calculate ROS activity levels for each condition. Data are shown as mean ± s.e.m., *n* = 3–5 animals, *p≤ 0.05. Statistical significance was calculated using one-way ANOVA with Neuman-Keuls post-test.

Total signal intensity measured using Maestro imaging showed that levels of ROS activity increased significantly after both mild and severe blast exposures at 24 h post-blast exposure. The activity levels were almost twice as high in the severe bTBI group versus naïve group (Fig 7). The change in brain ROS levels at different times post-exposure was determined in animals exposed to mild bTBI. The results show increase in ROS activity over time, with a peak surge occurring during the first 48 h, before reaching a steady level by 72 h (Fig 7).

#### Immunohistochemical analysis of brain tissues post-blast exposure

**Fig 8:**
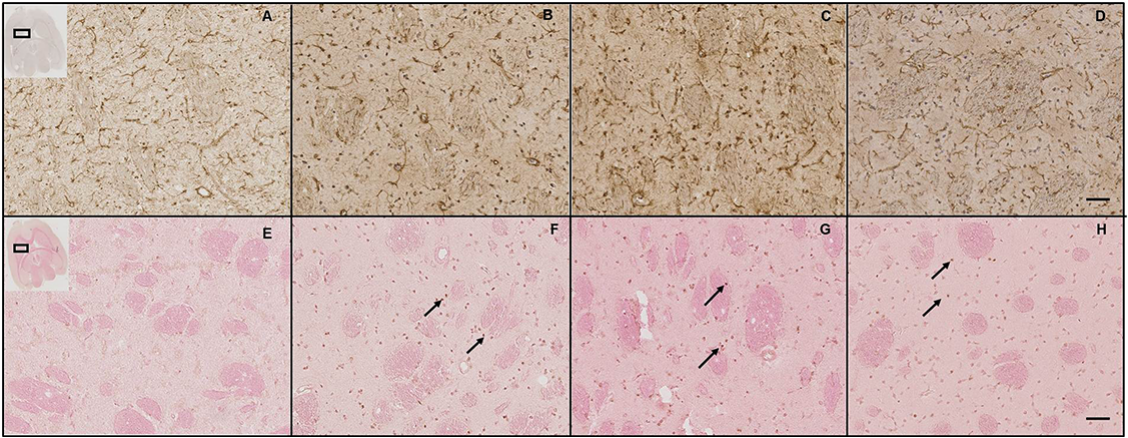
Immunohistochemical analysis of brain sections following mild bTBI. **A)** GFAP expression in brain sections at different time points post-injury. Increased expression of GFAP is seen in the thalamus of bTBI animals. Brain sections taken from naïve (A), and at 4 (B), 7 (C) and 11 (D) days post-exposure. **B)** Apoptosis at different time points post-injury. Apoptotic cells (arrows) are observed in several areas (thalamus shown) of the mild-bTBI brains. Brain sections taken from naïve (E), and at 4 (F), 7 (G) and 11 (H) days post-exposure. Scale bar: 50 μm.

Immunohistochemical analysis of the brain sections following blast exposure shows apoptosis and astrogliosis in the bTBI brains. We find an increased expression of GFAP in the thalamus at 4 and 7 days as compared to that in naïve brains, indicating the occurrence of astrocytosis post-blast exposure (Fig 8). At 11 days we see much less GFAP expression than observed on 4 and 7 days post- injury. We also see apoptosis at 4, 7 and 11 days post mild-bTBI (Fig 8) with numerous apoptotic cells scattered all over the brain tissue. The increase in GFAP expression is in the same tissue and area where we see significant amount of apoptosis as mentioned above.

## Discussion

Accurate replication and modeling of battlefield injuries associated with blast waves is an indispensable task in military-relevant bTBI research. Most shock-tube models currently employed to study bTBI exhibit significant diversity and notable disparities in the overall shock-tube design, membrane material, gases/explosives used, animal orientation, blast methodology, physics and parameters, specifically the peak overpressures and duration of exposure, making it difficult to analyze and compare results from extant literature [15]. Shock-tube designs that are capable of delivering repeatable and consistent peak overpressures and adequately mimic the fundamental parameters of blast-associated shock wave injuries are of critical importance in studying the mechanisms by which these waves cause neuronal and cerebrovascular damage.

In this study, we demonstrated the development and characterization of a laboratory-based shock-tube model able to produce a range of blast pressures to generate mild, moderate, and severe bTBI in rats (Fig 1). Characteristics of blast-wave profiles generated from all driver sizes showed steady consistencies in peak pressures and positive pulse durations (Table 1). The peak overpressures could be adjusted over an extensive range (20–130 psi) with positive pulse durations of 2–4 ms by selection of differently sized drivers (Table 1, Fig 3), and corresponding pressure-time profiles of the generated wave forms being identical to free-field blast waves (Fig 2).

Broadly categorized, shock tubes employ either expansion of compressed gases, deflagration of gas fuels, or detonation of explosives to generate a pressure wave [31,32]. Often, a performance goal of a shock tube generator is a Friedlander waveform, a pressure-time curve characteristic of an explosion in free field, with no proximate surfaces interacting with the wave [21]. Both compression-driven and detonation-driven shock tube designs are unable to replicate an ideal Friedlander waveform as apart from creating a primary shock wave, they generate complex pressure waves. These include reflected waves, rarefaction waves, Mach stem and turbulent jet waves that further transfer momentum and kinetic energy to the specimen/animal causing exacerbated blast injuries [33]. In compression-driven shock tubes, the initial shock wave is followed by a second pressure wave due to what is sometimes called the “jet effect” of expanding gases following the shock front. This jet effect applies a second loading to the animal transferring additional momentum, thus possibly inducing injury via mechanisms not corresponding to real blast loading [31]. Compression-driven shock tube designs are also known to create longer pressure durations which are often not observed in realistic scenarios. Detonation-driven shock tube designs are usually associated with large overhead expenses, facilities and safety concerns which involve storage and use of explosives, thus often unsuitable to be utilized in a laboratory setting or for animal studies [21,34]. The shock tube design in the present study has been developed and optimized to assess the outcomes of blast exposures on rats. Our shock tube has a simple modular design, is easy to setup and runs on commercially available hardware and software to generate a streamlined blast wave with precise blast overpressures.

A blast wave is minimally characterized by two values: the peak blast pressure and the duration of overpressure, both of which are known to be instrumental in causing physiological damage [35]. The peak pressure of a blast wave is reached in a few microseconds, appearing to be almost instantaneous on the scale of most pressure-time plots. Following the shock front, pressure decreases nearly exponentially over a time period that may be less than a millisecond to several hundred milliseconds, depending on the source of the blast and the distance of the subject from the center of the blast. Different explosive threats have different ranges of peak pressures and durations in air blasts. IEDs, hand grenades, and some mortars have a positive phase duration of 0.2–1 ms at about 1 m from the center of the blast, depending on the amount of explosive; about 2 ms for distances of 2–3 m and about 4 ms for distances of 10–100 m. The peak pressure depends on the distance of the subject from the center of the blast; at the center, peak pressure is a maximum of approximately 70 MPa [36]. At 1 m from the center of the blast, the peak pressure can still be lethal to humans and may range from 0.7 MPa (100 psi) for a hand grenade (equivalent to 0.23 kg TNT) to 7.0 MPa (1000 psi) for a 155-mm high-energy projectile (equivalent to 10.8 kg TNT).

The risk of blast injury depends on both the peak pressure and the duration of the blast wave. Areas of interest for exposures leading to bTBI by thoracic and acceleration mechanisms have also been published [5]. Peak pressures of approximately 140–900 kPa (20–130 psi) in the present study were produced with overpressure (positive phase) durations of 2–4 ms. This range is consistent with possible exposures caused by an IED explosion [29,37]. It is in the range of peak pressures and durations that have been associated with both acceleration and thoracic mechanisms of traumatic brain injury in humans. We did not see significant brain damage at pressures below 20 psi, which is in agreement with recent findings stating that pressures greater than 20 psi are required to induce bTBI, as the unprotected animal body can survive a relatively high shock pressure in the free field without experiencing barotraumas [33]. The venting jet flows of our device are very small [21], do not introduce quasi-steady shocks and transfer little momentum to the animal [38]. Placing the animals in front of the shock tube in our experiments, we have seen no instances of any ablative action or penetrating damage from jet impingement. The volume of air pushed out of the front of the tube is less than the volume of air originally contained in the driven section of the tube. In contrast to other studies in which animal mortality is prevalent [20,39,40], we have noted 100% survival of rats, with no early or late mortality in all studies of mild, moderate, and severe bTBI.

BBB damage is often implicated in causing secondary damage from bTBI by further aggravating the primary injury, with rapid but prolonged onset occurring within 24 h and lasting up to a few days to weeks [41–43]. In our study, when tested at 24 h following the blast exposure, we found increased vascular leakage with increase in peak overpressures of the shock wave (Fig 4A) and mapping of the brain slices for vascular leakage using optical signal intensity showed that different brain areas reacted differently to the blast exposure (Fig 5A and 5B). This could be attributed to the different mechanics and densities of tissue between the white- and gray-matter interface and/or the fluid-tissue interfaces, which ultimately causes different areas of the brain to be affected differently by blast forces, causing either acceleration, deceleration, or shearing of the underlying tissue [44]. Moreover, coup-contrecoup injury and the presence of ridges and protuberances in the base of the skull can also contribute to lacerations and contusions in composition of the brainstem and cerebral cortex, which can disrupt the functioning of neurons and axonal fibers [45].

Although the exact mechanism that propagates the primary bTBI is not known, unexpected commotion in streamlined blood flow seems to occur from the sudden over pressurization caused by the blast wave [4,5]. As a result, microscopic damage ensues in the blood vessels, which causes and further exacerbates the primary injury, possibly resulting in global cerebrovascular insults. In a recent study by Chen et al [46], they demonstrated that the kinetic energy of a blast wave causes rapid displacement of blood leading to a large volumetric surge which may further propagate cerebrovascular damage to the BBB. According to this hypothesis, which has been communicated previously [5,47,48], this universal damage to the blood vessels and BBB could be the main causes for mediating primary blast trauma into secondary neuronal dysfunction and later into cognitive and psychiatric disorders (PTSD). The BBB is a selectively permeable barrier unique to blood vessels and capillaries of the brain; some level of blast-wave exposure causes it to lose its integrity, which ultimately disrupts the homeostasis of electrolytes in the brain, thus aggravating the cerebral edema and neuronal inflammation arising from the primary injury [43,49,50]. Extravasation of IgG in cases of BBB breakdown associated with bTBI have been reported in literature previously [24,40,41], and our qualitative assessment of the systemic IgG adheres to similar findings (Fig 4B). Comparing the distribution of IgG leakage to our EB vascular disruption results, we see overlapping areas of the brain with leakage seen specifically in the hippocampus, thalamus and striatum by both the methods. An increased permeability of the BBB offers a passage for passive fluid movement within the brain, which can further exacerbate the rapid edema formation that is often associated with traumatic brain injury, usually occurring in the first few hours to days [43,51,52]. This theory has been supported by our study, wherein we demonstrate a tangible increase in the brain water content in the severe blast injury group determined as early as 3 h post-blast exposure (Fig 6). This data suggest that BBB disruption could be an early event following the blast-exposure. Given the selective function and specialized structure of the BBB to maintain brain homeostasis, further understanding of the mechanism of BBB disruption [53] could help in developing therapeutic strategies aimed at protecting it, which could thus minimize the effect of blast-induced damage [42].

Our results showed increased ROS activity in mild and severely injured animals, with sustained and escalated ROS levels observed up until 72 h post-mild blast exposures (Fig 7). It is known that ROS induce oxidative stress causes cell membrane, protein and DNA damage that can trigger a cascade of degenerative events including inflammation and neuronal cell death [32,54–56]. In addition, ROS can cause further disruption of the BBB [57,58], which could initiate a series of secondary insults such as hemorrhage, increased intracranial pressure, excitotoxicity, ionic imbalances, and hematomas [43]. Further studies are needed to understand the role of ROS in vascular damage as well in cellular apoptosis to develop efficient therapeutic intervention to alleviate the damage caused by bTBI [59].

Most of our conclusive information on bTBI comes from a combination of findings observed in military and civilian populations and laboratory based animal studies. Based on the physiological injury parameters observed in all cases, i.e. hematoma, hemorrhage, edema, vasospasm, disruption of the BBB and oxidative stress, we designed our study to evaluate the markers of interest that have been robustly associated with bTBI characteristics [1,60]. From our current results, we have established that a single exposure to a blast wave can cause substantial vascular injury and sustained oxidative stress, which can further propagate edema, inflammation and cell death. In recent conflicts, repeated exposures of military personnel to low levels of blast waves from IEDs are believed to account for majority (~75%) of the reported TBIs [61].

It has been known that TBI due to blast waves is a complex pathophysiological process that leads to deficits in motor, sensorimotor, and cognitive functions [15,59]. Several studies have suggested involvement of ROS not only in BBB dysfunction [57] but also their role in membrane lipid peroxidation, DNA/protein damage which correlate to the intensity of post-injury pathophysiological changes [62]. In this regard, we plan to determine the role of antioxidants such as superoxide dismutase (SOD) and catalase. In our previous studies, we have shown that antioxidant (superoxide dismutase and catalase)-loaded biodegradable nanoparticles (NPs) are effective in protecting human neurons from hydrogen peroxide-induced oxidative stress whereas enzymes alone or PEGylated enzymes were ineffective [63,64]. Further, we have shown that antioxidant-loaded NPs are effective in protecting BBB and preventing neuronal damage, resulting in better neurological recovery and survival of animals than untreated animals in a middle cerebral artery occlusion model of stroke in rats. The efficacy of the antioxidant-loaded NPs was determined by their ability to neutralize excessive production of ROS following ischemia-reperfusion [65]. Since we see increased ROS activity in the brain in blast-exposed animals, we anticipate that an effective delivery of antioxidant-loaded NPs to the brain though the disrupted BBB could mitigate the deleterious effects of the ROS-mediated cascade of degenerative events following blast-exposure.

## Conclusions

We designed a laboratory-scale shock tube capable of producing a wide range of peak pressures (20–130 psi) to create bTBI in rats. The blast waves produced by the shock tube have clinically relevant shock wave profiles, as typically seen in battlefield conditions. The increased and sustained levels of ROS seen in animals with bTBI could be a contributing factor in BBB leakage and propagating further neuronal damage. Our future experiments are aimed at neutralizing the deleterious effects of ROS that may effectively inhibit the cascade of neurodegeneration.
